# A note of thanks to referees in 2011

**Published:** 2011

**Dementia & Neuropsychologia** finishes the fifth year of publication and we would like to thank our referees for sharing their expertise with us. The high quality of constructive criticisms and dedication of all our reviewers are highly appreciated by both the editors and the authors.

Alessandro Ferrari Jacinto (São Paulo, SP)

Almir Ferreira de Andrade (São Paulo, SP)

Amaury Batista da Silva (Recife, PE)

Ana Luiza Rosas (São Paulo, SP)

Carlos Roberto de Mello Rieder (Porto Alegre, RS)

Cláudia Sellitto Porto (São Paulo, SP)

Dalva Poyares (São Paulo, SP)

Daniel Fuentes (São Paulo, SP)

Delson José da Silva (Goiânia, GO)

Élia Baeta (Madeira, Portugal)

Eliane Correa Miotto (São Paulo, SP)

Eliane Mayumi Kato-Narita (São Paulo, SP)

Elizabeth Frohlich Mercadante (São Paulo, SP)

Eneida Mioshi (Sydney, Australia)

Fabio Henrique de Gobbi Porto (São Paulo, SP)

Florindo Stella (Rio Claro, SP)

Francisco de Assis C. do Vale (São Carlos, SP)

Gilberto Ney Otoni de Brito (Rio de Janeiro, RJ)

Hae Won Lee (São Paulo, SP)

Jerson Lacks (Rio de Janeiro, RJ)

Jerusa Smid (São Paulo, SP)

Joana Bisol Ballardin (Porto Alegre, RS)

José Luiz Dias Gherpelli (São Paulo, SP)

Jose Marcelo Farfel (São Paulo, SP)

Leandro Malloy Diniz (Belo Horizonte, MG)

Leonardo Ferreira Caixeta (Goiânia, GO)

Lilian Schafirovits-Morillo (São Paulo, SP)

Lúcia Aparecida Bressan (Ribeirão Preto, SP)

Luis Felipe Berchielli (São Paulo, SP)

Maira Tonidandel Barbosa (Belo Horizonte, MG)

Marcelo E. Bigal (São Paulo, SP)

Marcia Maria Pires Camargo Novelli (Santos, SP)

Marcio Luiz Figueredo Baltazar (Campinas, SP)

Maria Joana Mader Joaquim (Curitiba, PR)

Mirna Lie Hosogi Senaha (São Paulo, SP)

Mônica Carolina Miranda (São Paulo, SP)

Mônica Sanches Yassuda (São Paulo, SP)

Mônica Zavaloni Scalco (São Paulo, SP)

Norberto Anizio Ferreira Frota (Fortaleza, CE)

Patricia Figueiredo do Vale (Ribeirão Preto, SP)

Paulo Caramelli (Belo Horizonte, MG)

Paulo Eduardo M. Carrilho (Cascavel, PR)

Paulo Henrique Ferreira Bertolucci (São Paulo, SP)

Paulo Mattos (Rio de Janeiro, RJ)

Renata Eloah de Lucena Ferretti (São Paulo, SP)

Rodrigo Bressan (São Paulo, SP)

Rosa Souza Cardoso Alves (São Paulo, SP)

Sandra Regina Schewinsky (São Paulo, SP)

Sérgio Luís Blay (São Paulo, SP)

Sonia Maria Dozzi Brucki (São Paulo, SP)

Tarso Adoni (São Paulo, SP)

Teresa Torralva (Buenos Aires, Argentina)

Tibor Rilho Perroco (São Paulo, SP)

Tin Po Huang (Brasília, DF)

Valeria Santoro Bahia (São Paulo, SP)

Ylmar Correa Neto (Florianópolis, SC)

